# Localisation of the SMC loading complex Nipbl/Mau2 during mammalian meiotic prophase I

**DOI:** 10.1007/s00412-013-0444-7

**Published:** 2013-11-28

**Authors:** T. Visnes, F. Giordano, A. Kuznetsova, J. A. Suja, A. D. Lander, A. L. Calof, L. Ström

**Affiliations:** 1 Department of Cell and Molecular Biology, Karolinska Institute, Berzelius väg 35, 171 77 Stockholm, Sweden; 2 Unidad de Biología Celular, Departamento de Biología, Universidad Autónoma de Madrid, 28049 Madrid, Spain; 3 Department of Developmental & Cell Biology, and the Center for Complex Biological Systems, University of California, Irvine, CA 92697 USA; 4 Department of Anatomy & Neurobiology, and the Center for Complex Biological Systems, University of California, Irvine, CA 92697 USA

## Abstract

Evidence from lower eukaryotes suggests that the chromosomal associations of all the structural maintenance of chromosome (SMC) complexes, cohesin, condensin and Smc5/6, are influenced by the Nipbl/Mau2 heterodimer. Whether this function is conserved in mammals is currently not known. During mammalian meiosis, very different localisation patterns have been reported for the SMC complexes, and the localisation of Nipbl/Mau2 has just recently started to be investigated. Here, we show that Nipbl/Mau2 binds on chromosomal axes from zygotene to mid-pachytene in germ cells of both sexes. In spermatocytes, Nipbl/Mau2 then relocalises to chromocenters, whereas in oocytes it remains bound to chromosomal axes throughout prophase to dictyate arrest. The localisation pattern of Nipbl/Mau2, together with those seen for cohesin, condensin and Smc5/6 subunits, is consistent with a role as a loading factor for cohesin and condensin I, but not for Smc5/6. We also demonstrate that Nipbl/Mau2 localises next to Rad51 and γH2AX foci. NIPBL gene deficiencies are associated with the Cornelia de Lange syndrome in humans, and we find that haploinsufficiency of the orthologous mouse gene results in an altered distribution of double-strand breaks marked by γH2AX during prophase I. However, this is insufficient to result in major meiotic malfunctions, and the chromosomal associations of the synaptonemal complex proteins and the three SMC complexes appear cytologically indistinguishable in wild-type and *Nipbl* ^*+/−*^ spermatocytes.

**Electronic supplementary material:**

The online version of this article (doi:10.1007/s00412-013-0444-7) contains supplementary material, which is available to authorized users.

## Introduction

The structural maintenance of chromosome (SMC) complexes regulate several aspects of chromosome dynamics during the eukaryotic cell cycle. The best characterised of these complexes is cohesin, which is necessary for normal sister chromatid cohesion and segregation. The chromosomal association of cohesin is governed by the evolutionary conserved loading complex, which consists of a heterodimer between the Nipbl and Mau2 proteins (Michaelis et al. 1997; Ciosk et al. 2000). This heterodimer loads cohesin prior to S-phase, as well as following genomic damage in the form of double-strand breaks (DSB) (Strom et al. 2004; Unal et al. 2004). In addition to their canonical involvement in cohesin loading, Nipbl and Mau2 have, in yeast, been suggested to also regulate the chromatin interactions of the two other known classes of SMC complexes, condensin and Smc5/6. Like the ring-formed cohesin complex, these complexes consist of a heterodimer of SMC proteins joined by a kleisin subunit and additional accessory factors (Hirano 2006). In the absence of the cohesin loader in *S* *accharomyces cerevisiae*, the chromosomal binding of both condensin and Smc5/6 is perturbed and reduced (D'Ambrosio et al. 2008; Lindroos et al. 2006). Furthermore, there appears to be a high degree of co-localisation between condensin and the cohesin loader (D'Ambrosio et al. 2008). On the other hand, in *Caenorhabditis elegans*, disruption of the NIPBL ortholog does not alter the binding pattern of neither condensin nor Smc5/6 (Lightfoot et al. 2011), and depletion of Scc2/4 in *Xenopus* egg extracts clearly affects cohesin but not condensin loading (Gillespie and Hirano 2004). Whether the condensin- or SMC5/6-related functions of the SMC loading complex are evolutionary conserved in mammals, have, to our knowledge, not been investigated.

Also during the generation of germ cells, all three classes of SMC complexes perform essential functions. During the meiotic prophase I, hundreds of DSBs are induced by Spo11 (Celerin et al. 2000). These are repaired and resolved by homologous recombination (Ahmed et al. 2010). Simultaneously, chromosomes are organised by the synaptonemal complex (SC), which forms a zipper-like structure that joins the two homologous chromosomes. The SC is defined by two lateral elements that are connected by transverse filaments. This structure facilitates proper DNA repair, synapsis, and the exchange of genetic material between homologous chromosomes. The cytological dynamics of chromosomes during prophase I allow its staging. Briefly, at the leptotene stage, DSBs are induced and chromosomes start to develop thin axial elements along them, marked by the Sycp3 protein. At the zygotene stage, repair of DSBs by homologous recombination using the sister chromatid as template is suppressed, instead the homologous chromosome is used. As a result, homologous chromosomes start to synapse, which is detected cytologically as longer, twinned lateral elements joined by transverse filaments of the SC. In mice, the regions close to the centromeres are the last to synapse. At the pachytene stage, homologous chromosomes are completely synapsed, including the centromeres and DSB repair is completed, resulting in crossing-over between homologous chromosomes. In mammals, this phase lasts for several days and the chromosomal structures are stabilised by a completely formed SC. In diplotene, synapsis and recombination is complete, and the homologous chromosomes start to desynapse but are still held together at chiasmata. The SC is then disassembled, starting with the transverse filaments. During these prophase I stages, different localisation patterns, reflecting their various DNA localisations, have been reported for the SMC complexes. Cohesin complexes, many of which are meiosis specific, are loaded onto chromosomes during (pre)leptotene and facilitate the assembly of the axial/lateral elements of the SC (Suja and Barbero 2009). Condensin I was reported to localise to nucleoli during pachytene and then appear at chromosome ends and chromatid cores in prometaphase I bivalents (Viera et al. 2007). Various meiotic localisations have been reported for the Smc5/6 complex which was found to be absent from meiotic chromosomes until late pachytene/diplotene, when it appeared on the sex body (Taylor et al. 2001). In addition, very recently, Smc5/6 was also shown to bind to chromosomal axes in one publication, as early as zygotene, and in two studies at chromocentres throughout the entire meiotic prophase (Verver et al. 2013; Gomez et al. 2013).

Nipbl and Mau2 interact with each other through their conserved N-terminal domains (Bermudez et al. 2012; Seitan et al. 2006) and associate with mitotic chromosomes from telophase until prophase, when the complex is excluded from chromatin (Watrin et al. 2006). Both Nipbl and Mau2 are essential due to their role in sister chromatid cohesion and segregation. Partial depletion of Nipbl reduces the association of cohesin to chromatin with deficient cohesion as a consequence (Seitan et al. 2006; Watrin et al. 2006). However, this is apparent only when the levels of cohesin or its loading complex are severely reduced. In several organisms, partial reduction of either confers defects in gene expression, development, and DSB repair, but not on sister chromatid cohesion or chromosome segregation (reviewed in Dorsett and Strom (2012)). In humans, mutation in the *NIPBL* gene results in the rare Cornelia de Lange syndrome (CdLS) (Krantz et al. 2004; Tonkin et al. 2004), a dominant autosomal disorder, affecting ~1:10,000 live born children and characterised by multiple organ system abnormalities, typical facial features, growth and mental retardation, upper limb defects and numerous other features (McNairn and Gerton 2008). At the cellular level, CdLS is characterised by transcriptional perturbations through mechanisms that are not well understood (Kawauchi et al. 2009; Liu et al. 2009, 2010). Cells from CdLS patients are also associated with radiation sensitivity (Vrouwe et al. 2007; Enervald et al. 2013).

NIPBL deficiency has also been shown to penetrate into meiotic defects in several organisms. In yeast, meiotic depletion of the Nipbl ortholog (Scc2) confers defects in sister chromatid cohesion, nuclear division and transcriptional dysregulation of multiple genes, including meiotic cohesin subunits (Lin et al. 2011). Mutation or downregulation of NIPBL orthologs displays multiple meiotic defects in *Arabidopsis thaliana* (Sebastian et al. 2009), and *Coprinus cinereus* (Seitz et al. 1996; Cummings et al. 2002), including failure to assemble the SC as well as DNA repair defects. During meiosis in *Drosophila*, the NIPBL ortholog co-localises with the SC and cohesin except at the regions close to the centromeres. Haploinsufficiency of NIPBL here results in premature disassembly of the SC (Gause et al. 2008), without affecting chromosome segregation or fertility. In *C. elegans*, a mutation in *Scc2* abolishes loading of cohesin, but not the other SMC complexes to chromosome axes, leading to multiple cytological defects and a failure to repair SPO11-induced breaks (Lightfoot et al. 2011). In all cases, several meiotic cytological or functional defects are observed in otherwise seemingly healthy, viable organisms, suggesting that the meiotic cell divisions are more sensitive to Nipbl/Mau2 dysregulation than the mitotic divisions. Finally, Nipbl was recently shown to bind chromosomal axes during zygotene in murine germ cells, where it co-localised with cohesin and components of the SC (Kuleszewicz et al. 2013). However, the role of Mau2 in meiosis has, so far, not been investigated, and little is known about if and in what way partial deficiency of the SMC loading complex Nipbl/Mau2 affects meiosis in mammals.

Here, we have determined the meiotic localisation of Nipbl and Mau2 in wild-type and *Nipbl* ^*+/−*^ mouse germ cells and their associations with the SC, cohesin, condensin and the Smc5/6 complex, as well as markers of DNA damage and repair.

## Materials and methods

### Animals

C57BL/6 and CD-1 wild-type mice were acquired from Charles River and maintained along with *Nipbl* ^+/−^ (Kawauchi et al. 2009), *Sycp1* ^−/−^ (de Vries et al. 2005) and *Sycp3* ^−/−^ (Yuan et al. 2000) mice according to regulations provided by the animal ethical committee of Stockholm Region North, which also approved the experiments (N416/10).

### Immunofluorescence

Preparation of testicular and ovarian nuclear spreads was performed according to methods for surface spreading of meiotic chromosomes described previously (Peters et al. 1997). We used the following antibodies and dilutions for the immunofluorescent detection of proteins: rabbit anti-Mau2 (Abcam #46906) 1:60, rabbit anti-H3K9me3 (Abcam #103226) 1:500, rabbit anti-Sycp3 (Liu et al. 1996) 1:200, guinea pig anti-Smc1β and anti-Stag3 (Kouznetsova et al. 2005) 1:200, rabbit anti-Smc6 (AbCam #18039) 1:200, rabbit anti-CAPG (Heale et al. 2006), 1:500 for immunofluorescence and 1:3,000 for Western blots, mouse anti-γH2AX (Millipore) 1:1,000, human anti-CREST 1:1,000, human anti-ACA 1:100, and rabbit anti-Smc3 1:500 (Abcam #9263). Guinea pig anti-Nipbl (SKEVQDKDKPLKKRKQDSY) and anti-Mau2 (WTDGPPPVQFQAQNGPNTS) were generated against the indicated peptides (Peptide Specialty Laboratories, Germany), and affinity-purified on columns coupled to the corresponding peptides and used at 1:200 for immunofluorescence or 1:1,000 for Western blots. All non-commercial antibodies not previously used on samples from mice showed a single band of the predicted size in Western blots (Supplementary Fig. 1). All stainings that compare genotypes were done using the same antibody dilutions, on slides prepared in parallel. The slides were viewed at room temperature using a Leica DMRA2 microscope. Images were captured with a Hamamatsu digital charge-coupled device camera C4742-95 viewed with Volocity software (PerkinElmer). Mouse embryonic fibroblasts (MEFs) were grown on coverslips. Before and 60 min after irradiation, cells were fixed in 4 % formaldehyde in phosphate-buffered saline (PBS) for 15 min at 22 °C. The slides were incubated in 0.1 % glycine/PBS for 30 min, blocked in 3 % BSA, 10 % goat serum and 0.05 % Triton X-100 in PBS and stained as described above.

### Extract preparation and Western blot detection

Testes from wild-type and *Nipbl* ^+/−^ animals were detunicated, torn into four to eight pieces and incubated with 0.125 % trypsin in a shaking incubator at 37 °C at 180 rpm for 15 min. After allowing debris to sediment, the supernatant was transferred to 10 % foetal bovine serum in PBS after being passed through a 70-μm sieve. After repeating the trypsin treatment on the sedimented material once, the single cells were pelleted at 1,000×*g* at 4 °C for 5 min and washed twice in 50 ml of ice-cold PBS. The cells were resuspended in PBS supplemented with 1 % NP-40, complete protease inhibitors, 1 mM phenylmethylsulfonyl fluoride (PMSF) and 1 mM dithiothreitol (DTT) and left on ice for 45 min when insoluble material was removed by centrifugation at 16,000×*g* at 4 °C for 30 min. The supernatant was aliquoted and frozen at −80 °C until use. A similar volume of extract was loaded in 4–15 % Mini-PROTEAN TGX Stain-Free Gels (BioRad), ran at 300 V for 30 min and transferred onto PVDF membranes in 25 mM Tris, 192 mM glycine, 10 % methanol and 0.05 % sodium dodecyl sulfate (SDS) at 30 V for 60 min. After blocking the membrane with 5 % dry milk in TBS-T, Nipbl and tubulin were detected using guinea pig anti-Nipbl, guinea pig anti-Mau2 and mouse anti-tubulin (Sigma) at 1:1,000 and 1:5,000 dilutions, respectively. Protein extracts from MEFs were prepared with standard procedures using lysis buffer (10 mM Tris–HCl pH 8.2, 5 mM MgCl_2_ and 0.1 % SDS supplemented with 1 mM PMSF, 10 mM DTT, 1× Protease Inhibitor Cocktail (Roche), 1 U DNase and 10 mg/ml RNase). Protein extracts were analyzed by SDS-polyacrylamide gel electrophoresis (PAGE) and Western blotting. For detection of Nipbl, Mau2 and Cap-G, 4–12 % Bis–Tris gels were run in 1× MOPS buffer (NuPAGE Invitrogen). Proteins were transferred to nitrocellulose membrane (Protean) in 1× transfer buffer (NuPAGE Invitrogen). Primary antibodies used were the same as for IF: guinea pig anti-Nipbl (1:1,000), anti-Mau2 (1:1,000) and anti-Cap-G (1:3,000).

### MEF preparation and colony formation assay

MEFs were prepared from E13.5 embryos and immortalised by serial passages essentially as described (Xu 2005). Radiation sensitivity was measured by colony formation assays. Briefly, twofold dilutions of MEFs were seeded in six-well plates (125–4,000 cells/well) and irradiated with 1, 3 or 5 Gy. Colonies were allowed to form during a 12-day incubation, after which they were fixed with methanol and stained with Giemsa (Sigma-Aldrich, GS500), according to the manufacturer's instructions.

### Analysis of meiosis in yeast

SK1 yeast cells with the genotype MATa/MATα, Ho::LYS2/ho::lys2, ura3/ura3, leu2/leu2, trp1/trp1, his3/his3 and lys2/lys2 were made Scc4 meiotic null by insertion of the pCLB2 promoter upstream of the SCC4 gene at its endogenous loci resulting in a strain with the following genotype: SK1 MATa/MATα, HO::LYS2/ho::lys2, ura3/ura3, leu2/leu2, trp1/trp1, his3/his3, lys2/lys2 and KAN::pCLB2::3HA::SCC4/KAN::pCLB2::3HA::SCC4. These cells were forced to undergo synchronous meiosis by release from pre-sporulation medium to sporulation medium at a cell density of 2–5 × 10^7^ cells/ml. Samples were taken at the indicated time points and fixed in 100 % ethanol. 4',6-Diamidino-2-phenylindole (DAPI)-stained nuclei were then counted. Protein lysates were prepared with TCA extraction. Proteins were separated by SDS-PAGE using NuPAGE Bis–Tris Gels and detected by Western blotting (Life Technologies), using mouse anti-HA (Roche) and rabbit-anti-cdc11 (Santa Cruz Biotechnology).

## Results

### Chromosomal localisation of Nipbl/Mau2 in prophase I spermatocytes

**Fig. 1:**
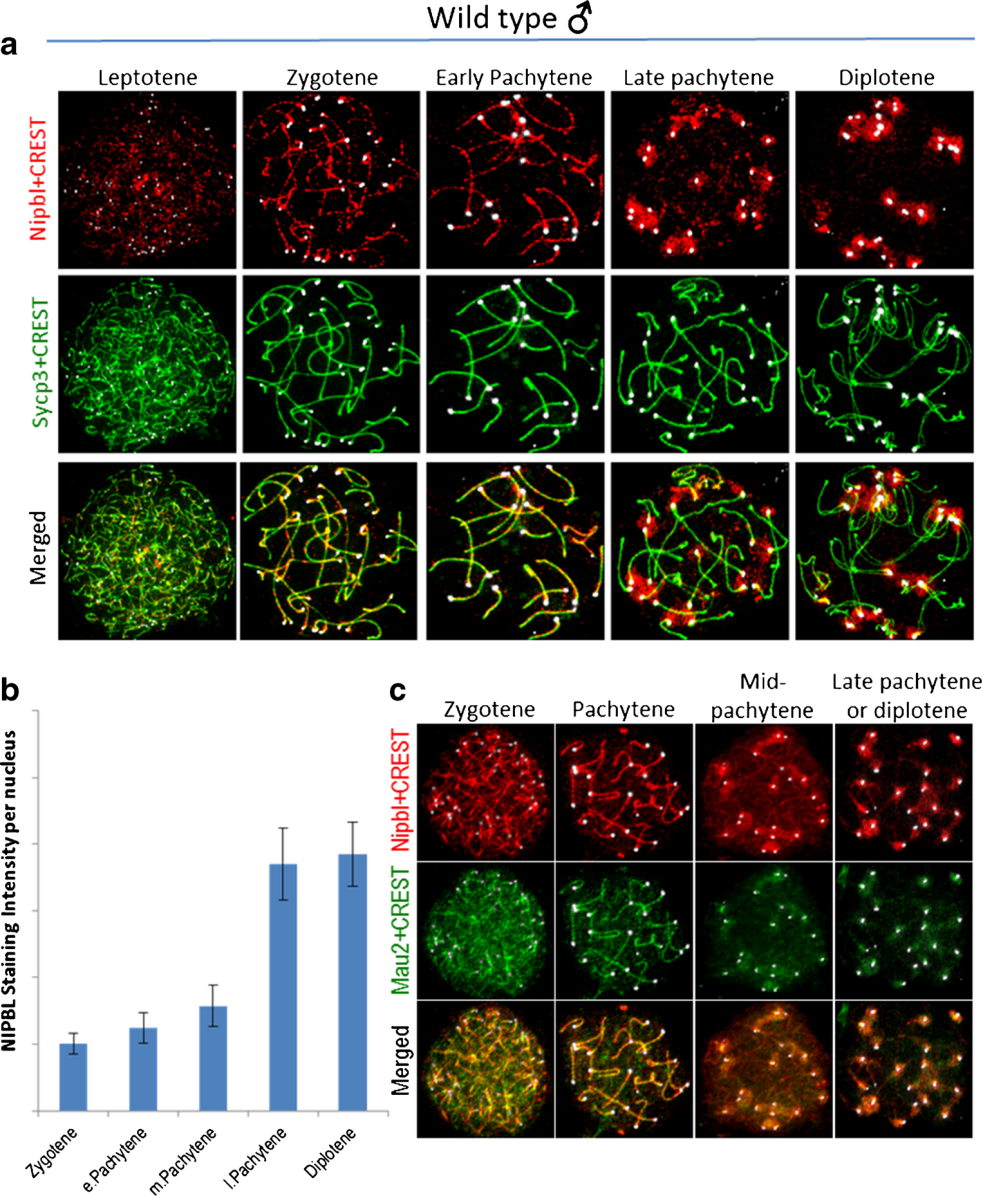
Chromosomal localisation of Nipbl/Mau2 in prophase I spermatocytes. **a** Testicular nuclear spreads were stained with rabbit anti-Sycp3 (*green*), guinea pig anti-Nipbl (*red*) and human anti-CREST (*white*), and images were staged according to established Sycp3 and CREST-staining patterns during prophase I. Nipbl was found on chromosomal axes from leptotene until mid-pachytene, when it translocated to chromocentres. **b** Using identical imaging settings, Nipbl staining of individual nuclei were quantitated, subtracting the background intensity of a neighbouring empty area. Nipbl staining was three- to fourfold more intense during late pachytene/diplotene stages than at earlier stages. **c** Testicular nuclear spreads, stained with rabbit anti-Mau2 (*green*), guinea pig anti-Nipbl (*red*) and human anti-CREST (*white*)

To examine the localisation of Nipbl/Mau2 during the mammalian meiotic prophase I, we prepared testicular spreads of spermatocytes derived from adult mice. The spreads were stained with antibodies against Sycp3, Nipbl and human anti-centromere serum (CREST). We then imaged cells and staged them according to the staining pattern of Sycp3 and CREST (Page and Hawley 2004). During leptotene, Nipbl was detected as small accumulations associated with chromosomal axes. In zygotene and pachytene stages, Nipbl staining along lateral elements was rather uniform, co-localising with Sycp3. However, unlike Sycp3, Nipbl did not stain the chromosomal axes continuously but rather unevenly with a punctuate pattern (Fig. 1a). In mid-pachytene, the signal of Nipbl along chromosomal axes gradually decreased, and we observed its accumulation at chromocentres, with a sharp increase in intensity in the region of the centromere itself. This relocation was associated with an accompanying intensification in total nuclear Nipbl staining that per nucleus was ~4-fold higher in late pachytene and diplotene than in zygotene, when imaged and quantitated under identical imaging settings (Fig. 1b). Post-prophase I, Nipbl appeared at centromeres, mostly co-localising with Sycp3, but also at pairs of spots suggestive of centrioles (Supplementary Fig. 2). Co-localisation of Sycp3 and Nipbl at centromeres vanished during anaphase I, whereas the signals at presumptive centrioles were still evident (Supplementary Fig. 2). Nipbl was also observed at interkinesis chromocentres, but not on metaphase II chromosomes. In early round spermatids Nipbl appeared at chromocentres, whereas in elongated spermatids a pair of dots were evident at the base (Supplementary Fig. 2).

A similar pattern of distribution during prophase I stages was observed for Mau2 when combining the guinea pig anti-Nipbl with a Mau2 antibody raised in rabbit (Fig. 1c). Similar to Nipbl, we observed Mau2 along elongating axial/lateral elements in leptotene/zygotene (Fig. 1c). Both Nipbl and Mau2 were distributed along chromosomal axes in a discrete, punctuate pattern in pachytene. Then, in mid-pachytene, both Nipbl and Mau2 migrated towards chromocentres in a highly synchronous manner. Essentially identical staining patterns and intensities were observed when co-staining with guinea pig anti-Mau2 and rabbit anti-Mau2 antibodies (data not shown). This demonstrates both the specificity of the antibodies and that Nipbl and Mau2 co-localise throughout prophase I. Moreover, this suggests that they do not perform independent functions during mammalian meiosis, at least not as can be cytologically discernible. Supporting this, ablation of *S. cerevisiae* Scc4 specifically during meiosis results in a near complete failure to undergo meiosis (Supplementary Fig. 3). Only a few percent of the cells managed to pass the first meiotic division, similar to what was observed for a Scc2 meiotic null strain (Lin et al. 2011).

### Distribution of Nipbl/Mau2 in prophase I oocytes

**Fig. 2:**
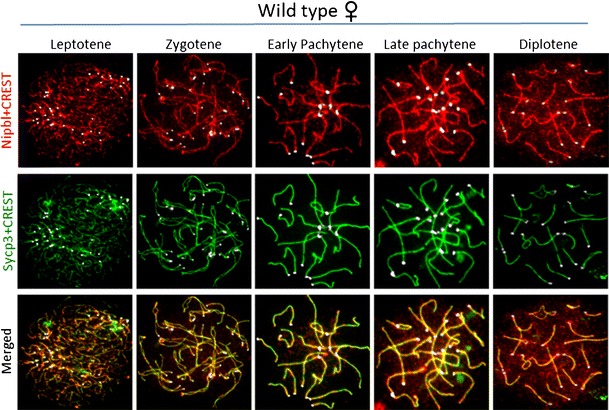
Chromosomal localisation of Nipbl/Mau2 in prophase I oocytes. Developing oocytes were isolated from female embryos at E16.5 to E19.5 and stained with rabbit anti-Sycp3 (*green*), guinea pig anti-Nipbl (*red*) and human anti-CREST (*white*). Images were staged through meiosis prophase I as in Fig. 1. Nipbl binds chromosomal axes throughout the meiotic prophase

During the early meiotic prophase in female embryos, harvested at embryonic age E16.5–E19.5, we observed an essentially similar distribution of Nipbl along chromosomal axes as in spermatocytes (Fig. 2). We detected both Nipbl and Mau2 (not shown) along elongating axial elements during leptotene. Nipbl/Mau2 bound strongly at regions close to the centromeres and weaker along the axial elements. During zygotene and pachytene stages, the Nipbl/Mau2 labelling was more uniform along lateral elements and showed strong co-localisation with Sycp3. However, unlike in spermatocytes, Nipbl/Mau2 retained on chromosomal axes in the later parts of pachytene and diplotene towards dictyate arrest, but was also present as a weak, diffuse staining in the nucleus.

### Nipbl/Mau2 co-localises with cohesin during early prophase I stages

**Fig. 3:**
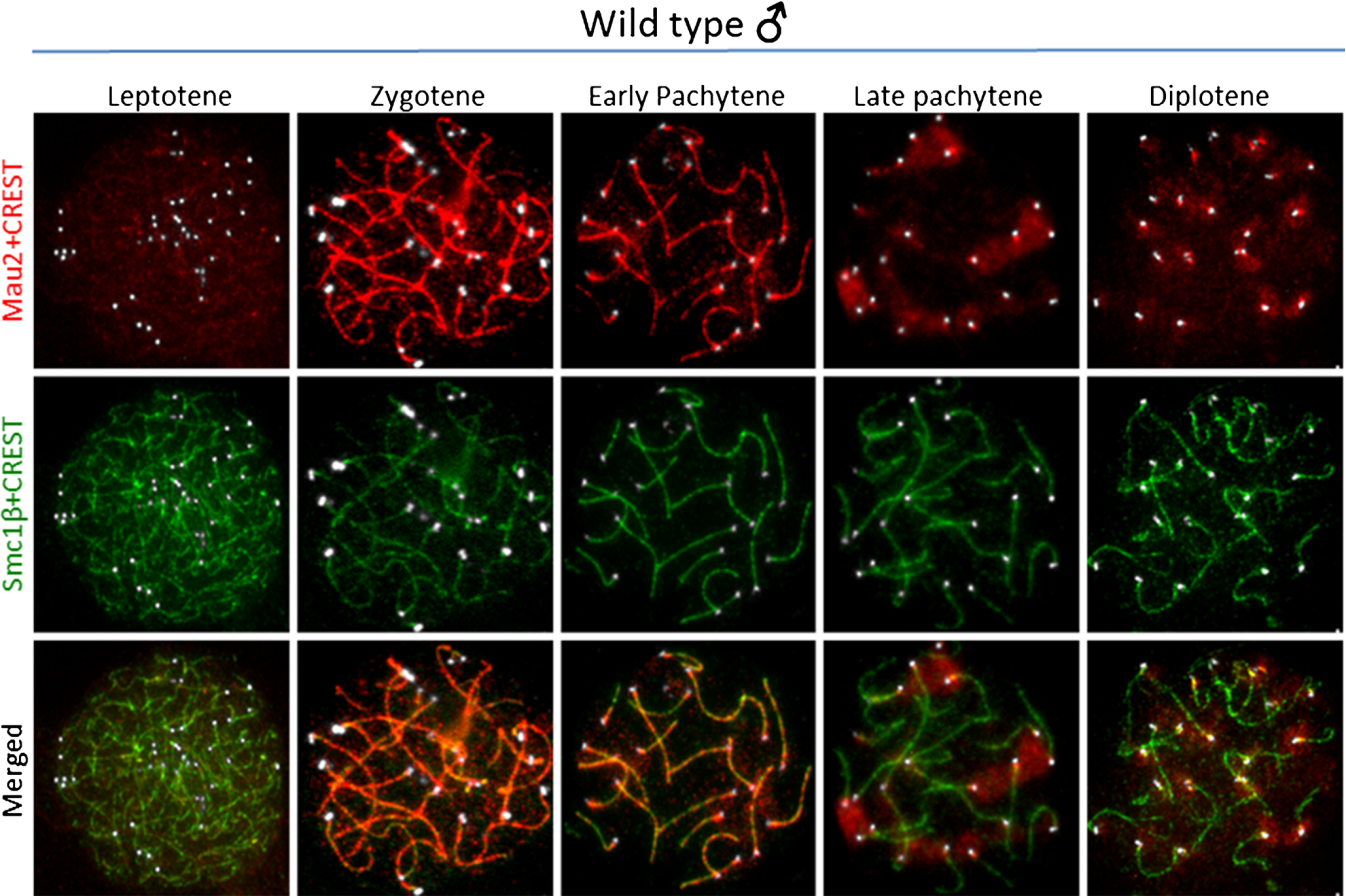
Mau2 and cohesin co-localise between zygotene and early pachytene in spermatocytes. Testicular nuclear spreads were stained with rabbit anti-Mau2 (*red*), guinea pig anti-SMC1β (*green*) and human anti-CREST (*white*). Images were staged through meiosis prophase I as in Fig. 1

**Fig. 4:**
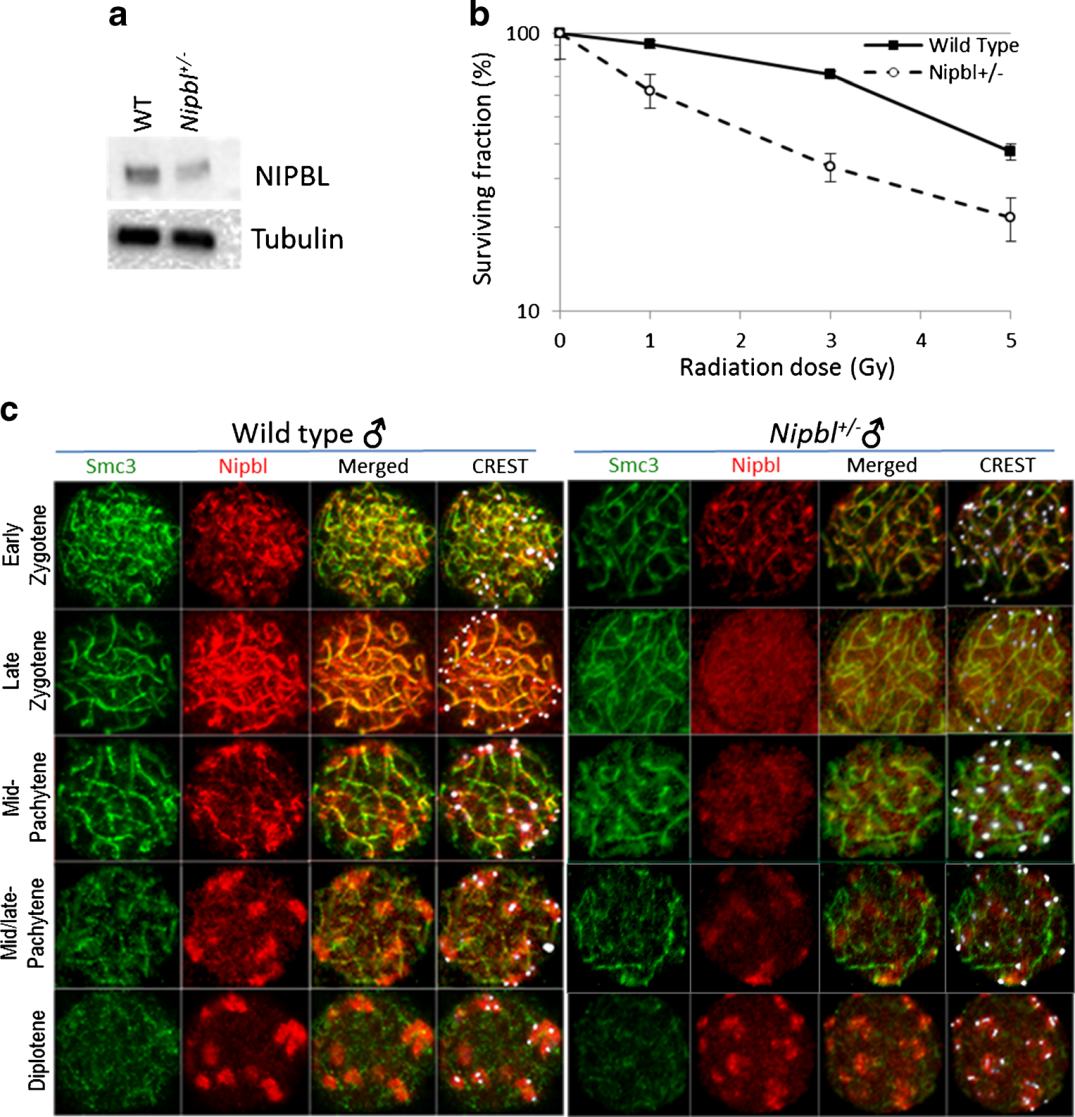
Reduced Nipbl spermatocyte expression and increased DNA damage sensitivity in *Nipbl* ^*+/−*^ MEFs. **a** Western blot showing reduced Nipbl expression in testis from *Nipbl* ^*+/−*^ animals using the same antibody as for immunofluorescence. **b** Wild-type and *Nipbl* ^*+/−*^ embryonic fibroblasts were exposed to the indicated radiation doses and allowed to form colonies. **c** Immunofluorescent staining of rabbit anti-Smc3 (*green*), guinea pig anti-Nipbl (*red*) and human anti-CREST (*white*) in prophase I spermatocytes from wild-type and *Nipbl* ^*+/−*^ mice. *Top row*: Nipbl was first detected in leptotene/zygotene stages in wild type or later in zygotene in *Nipbl* ^*+/−*^ spermatocytes. *Second row*: In late zygotene, Nipbl appeared to be lost from axial elements in *Nipbl* ^*+/−*^ spermatocytes. *Middle row*: Mid-pachytene spermatocytes displayed accumulation of Nipbl on chromocentres in both wild type and *Nipbl* ^*+/−*^, and Smc3 binding to chromosomal axes appeared normal. *Fourth row*: In late pachytene/diplotene spermatocytes, Nipbl was fully localised to chromocentres. *Bottom row*: Diplotene spermatocytes displaying progressively weaker Smc3 staining and full labelling of chromocentres by Nipbl. Images were staged through meiosis prophase I as in Fig. 1

To investigate whether Nipbl/Mau2 performed a similar function for cohesin loading in mouse meiosis as in mitotic cells, we co-stained spermatocytes with antibodies against cohesin subunits and Nipbl/Mau2 (Figs. 3 and 4c). While the meiosis-specific cohesin subunit Smc1β could be detected on elongating axial elements during early leptotene, similar to Sycp3, there was little co-localisation with its loading complex represented by Mau2, which was limited to a few foci and a rather diffuse staining all over the nuclear area (Fig. 3), similar to that described for Nipbl above. The highest degree of co-localisation was seen in zygotene, and by early pachytene, when several stretches of chromosomal axes free of Mau2 could be observed. By mid-pachytene cohesin was still found on chromosomal axes, whereas Nipbl/Mau2 had completely translocated to chromocentres. The difference in localisation between cohesin and its loading complex at this stage suggests that if the binding of Nipbl/Mau2 to chromocentres is functionally significant, then this function is unrelated to the loading of cohesin.

### Cohesin loading to chromosomal axes appear normal in *Nipbl*^*+/−*^ spermatocytes

To investigate whether reduction of the level of Nipbl would influence the recruitment of cohesin to meiotic chromosomes, we obtained *Nipbl* ^+/−^ mice (Kawauchi et al. 2009). First, we ascertained that Nipbl expression was reduced at the protein level (Fig. 4a and Supplementary Fig. 1). Indeed, expression was reduced with ~30 %, indicating that *Nipbl* heterozygousity is somewhat compensated by expression from the wild-type allele in mouse testis, similar to that previously reported for *Nipbl* ^*+/−*^ MEFs (Kawauchi et al. 2009). This reduction, however, was sufficient to confer radiation sensitivity in somatic cells (Fig. 4b), suggesting that a minor reduction in Nipbl is enough to induce DSB repair deficiency, similar to CdLS patient cell lines (Enervald et al. 2013; Vrouwe et al. 2007). A similar degree of reduction in Nipbl protein was also observed in *Nipbl* ^*+/−*^ spermatocytes, where it was readily apparent that Nipbl staining was substantially weaker than in the wild type (Fig. 4c). Chromosomal axes in zygotene and pachytene were harder to discern, and we observed an increase in general nuclear background staining. However, the same spatiotemporal distribution of Nipbl during prophase I was evident. Similar as in wild type, Nipbl was first detected on chromosomal axes during zygotene (Fig. 4c). At the late zygotene/early pachytene stage, Nipbl faded from the axes and stained the whole nucleus diffusely. Then, as in wild-type cells, Nipbl translocated to chromocentres where it bound for the remainder of prophase I. Similar observations were also made for Mau2 in *Nipbl* ^*+/−*^ spermatocytes (Supplementary Fig. 4). Thus, it appears that the association of Nipbl with chromosomal axes is sensitive to small changes in expression. Despite this, we could not observe any loading defect for cohesin, here exemplified by staining of Smc3 and Stag3 (Fig. 4c and Supplementary Fig. 5) (Barbero 2011). Cohesin assembled along lateral elements of the SC and showed the same localisation pattern as in wild type in the later stages of prophase I, even after Nipbl was removed from chromosomal axes. This is consistent with a model in which Nipbl functions as a cohesin loader, but is not required to maintain cohesin binding at chromosomal stages later than zygotene or early pachytene. Moreover, it is clear that the structure of the SC, once it is assembled in zygotene, is maintained even in the virtual absence of Nipbl along chromosomal axes (Fig. 4c).

### Influence of the SC components Sycp1 and 3 on Nipbl/Mau2 localisation

**Fig. 5:**
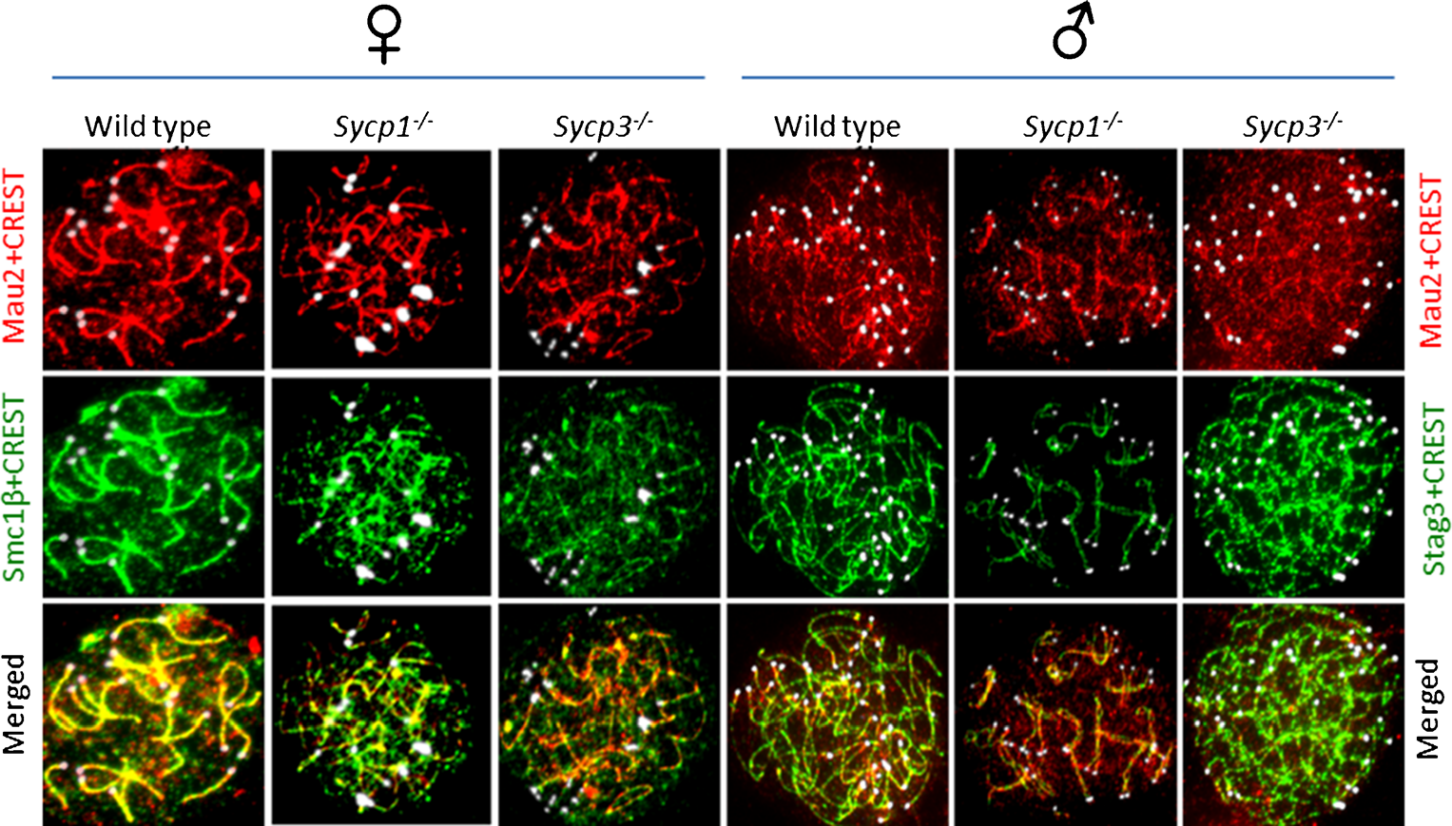
Influence of the SC components Sycp1 and 3 on Nipbl/Mau2 localisation. Nuclear spreads of embryonic ovaries or testes stained with rabbit anti-Mau2 (*red*), guinea pig anti-Smc1β (*green*, *left*), guinea pig anti-Stag3 (*green*, *right*) and human anti-CREST (*white*) in wild-type and *Sycp1* ^*−/−*^ or *Sycp3* ^*−/−*^ mice as indicated. Germ cells from *Sycp1* ^*−/−*^ and *Sycp3* ^*−/−*^ are arrested in a pre-pachytene state. Wild-type pachytene oocytes (*left*) and zygotene spermatocytes (*right*) are shown for comparison

Since Nipbl/Mau2 co-localised with Sycp3 at chromosomal axes, we investigated whether the absence of the SC components Sycp1 and Sycp3 would lead to defects in the binding of Nipbl/Mau2. For this, we employed male and female germ cells derived from mice carrying homozygous deletions in *Sycp1* and *Sycp3* genes (de Vries et al. 2005; Yuan et al. 2000). During the male prophase I, disruption of the SC activates the pachytene checkpoint, leading to apoptosis (Yuan et al. 2000). As a consequence, most germ cells from these animals assume a zygotene/early pachytene-like state. In both male and female *Sycp1* ^*−/−*^ germ cells, we could observe Nipbl/Mau2 binding, where chromosomal axes were visualised by Smc1β and Stag3 (Fig. 5), although the staining was considerably more diffuse than in wild-type zygotene nuclei. In *Sycp3* ^−/−^ germ cells, Nipbl/Mau2 bound to chromosomal axes even more weakly than in *Sycp1* ^*−/−*^, although the chromosomal axes were also considerably more rudimentary. Regardless, these results indicate that these proteins are still clearly able to bind to rudimentary chromosomal axes in the absence of either the axial elements or transverse filaments of the SC.

### Relative distributions of Nipbl/Mau2 and the SMC complexes condensin I and Smc5/6 in wild-type and *Nipbl*^*+/−*^ spermatocytes

**Fig. 6:**
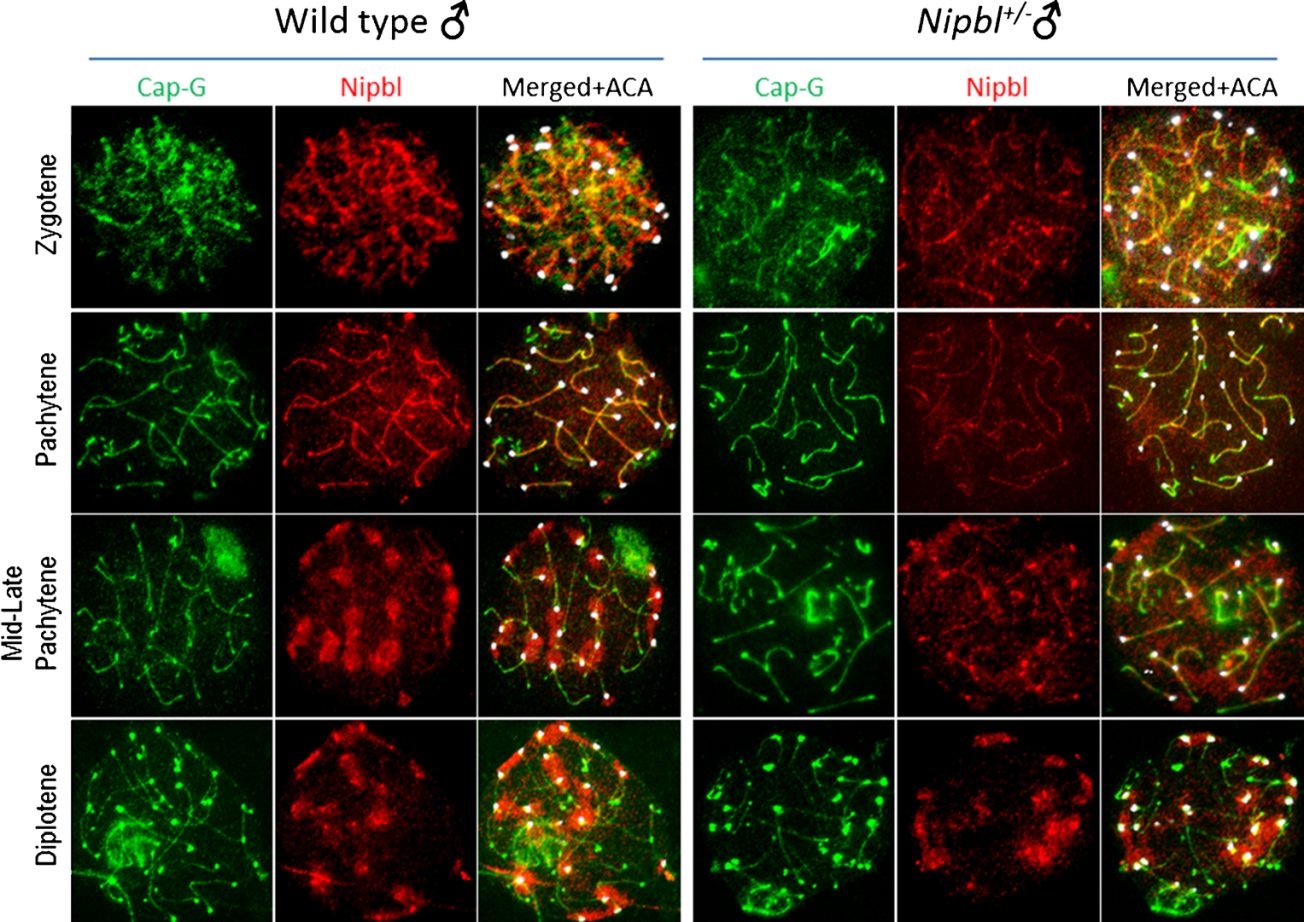
Nipbl and condensin I co-localise between zygotene and mid-pachytene in spermatocytes, but condensin staining is not affected by Nipbl haploinsufficiency. Testicular nuclear spreads of wild-type and *Nipbl* ^*+/−*^ spermatocytes were stained with rabbit anti-Cap-G (*green*), guinea pig anti-Nipbl (*red*) and human anti-CREST (*white*)

**Fig. 7:**
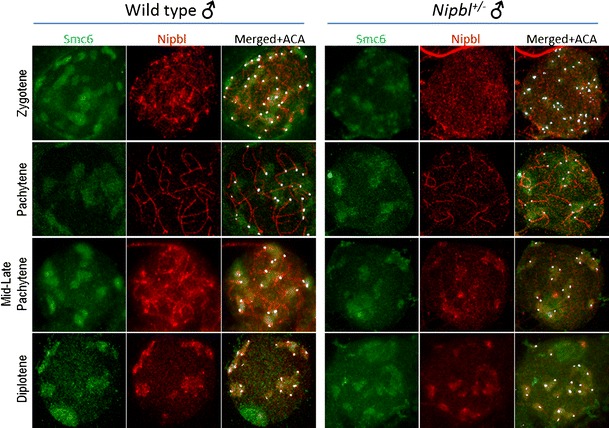
No apparent co-localisation between Nipbl and Smc5/6 in spermatocytes. Testicular nuclear spreads were stained with rabbit anti-Smc6 (*green*), guinea pig anti-Nipbl (*red*) and human anti-ACA (*white*)

In wild-type mouse spermatocytes, the distribution of condensin I, represented by the Cap-G subunit, appeared as short stretches of thread-like signals in leptotene/zygotene (Fig. 6). Similar to cohesin and Nipbl/Mau2, condensin I was found along the SC lateral elements in zygotene and pachytene spermatocytes. Then, during pachytene, condensin was gradually lost from chromosomal axes, but appeared to remain at chromosome ends. In diplotene, condensin I was still present along desynapsing lateral elements and at the sex body. Unlike cohesin and condensin, we could not detect the Smc5/6 complex at chromosomal axes. Instead, Smc6 was found at chromocentres (Fig. 7), where it stayed through the entire meiotic prophase in spermatocytes. The difference in timely association of Smc6 and Nipbl with chromocentres excludes a role of Nipbl as chromatin loader of Smc5/6 at least at these positions. Co-localisation with Nipbl/Mau2 was only observed at the sex body during these prophase I phases. In spermatocytes from *Nipbl* ^*+/−*^ animals, we detected no quantitative or qualitative difference in condensin or Smc5/6 staining compared with wild type, indicating that reduction in Nipbl protein level does not affect loading of any of the SMC complexes in mouse spermatocytes (Figs. 6 and 7).

### Nipbl/Mau2 does not co-localise with γH2AX or RAD51

**Fig. 8:**
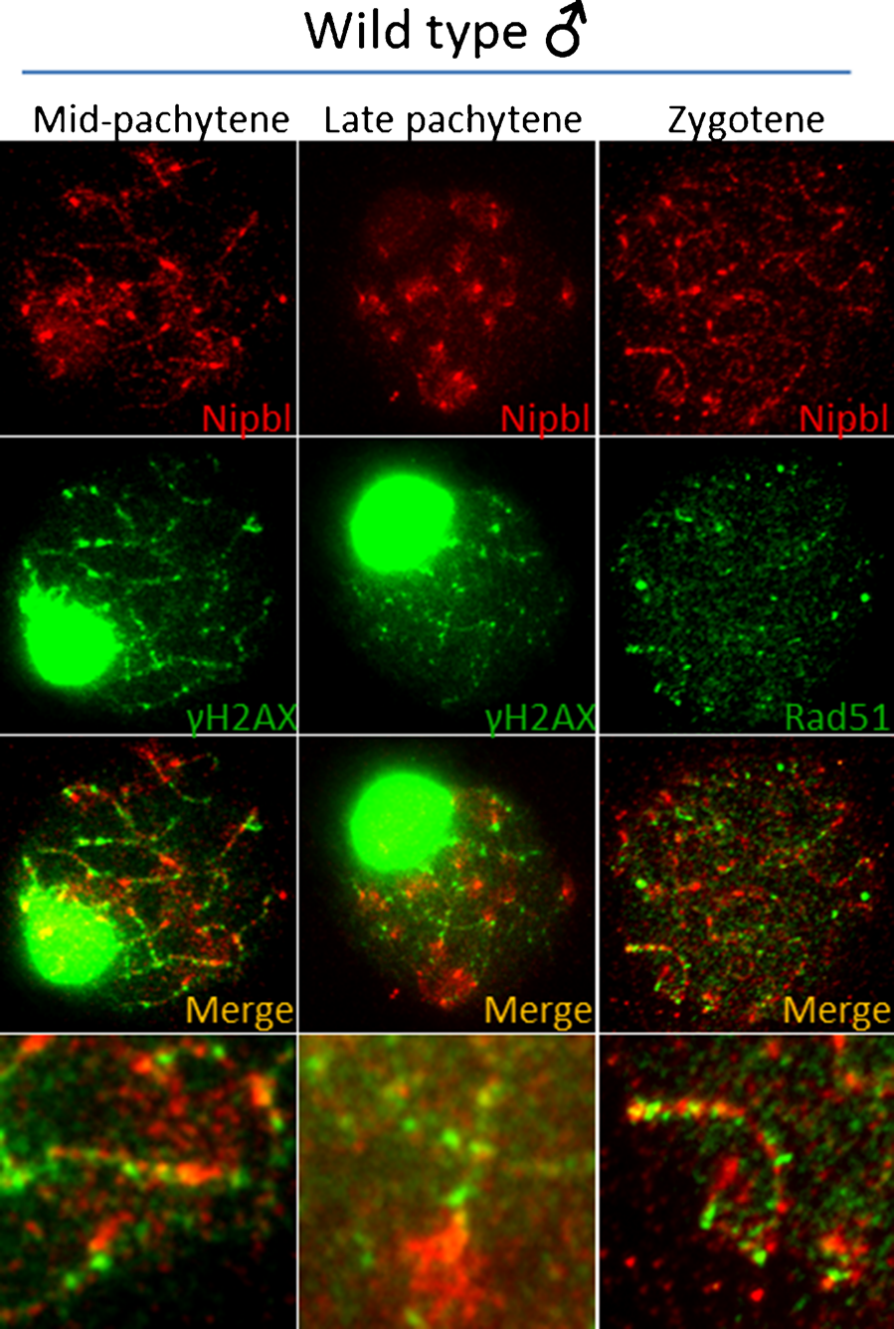
Nipbl does not co-localise with markers for DNA damage and repair. Testicular nuclear spreads were stained with guinea pig anti-Nipbl (*red*), mouse anti-γH2AX (*green in first three columns*) and mouse anti-RAD51 (*green in fourth column*)

Since it is firmly established that cohesin and Nipbl are important for HR-based DSB repair, and that meiotic DSBs are repaired via HR, we next wanted to determine whether we could detect Nipbl/Mau2 in their proximity, using well-characterised markers for DSBs, such as γH2AX and Rad51. γH2AX is phosphorylated in response to Spo11-induced DSBs in leptotene and zygotene and presents itself as a pan-nuclear staining during these stages. As DSBs are repaired, this staining fades gradually and in pachytene γH2AX is seen as a strong signal on the sex body, as well as relatively weak staining along chromosomal axes and chromosomal loops, thought to represent sites of ongoing DSB repair (Chicheportiche et al. 2007). During the course of the gradual DSB repair, detected as small speckles and foci of γH2AX along chromosomal axes, we could detect Nipbl/Mau2 in small stretches between these axial γH2AX speckles rather than co-localised (Fig. 8). While we cannot rule out binding of Nipbl/Mau2 to DSBs, it appears that the bulk of Nipbl/Mau2 associates to chromatin independently of DSB repair. This seems to be the case also in both wild-type and *Nipbl* ^*+/−*^ MEFs, where we were unable to detect Nipbl at γH2AX repair foci induced by irradiation (Supplementary Fig. 6). Similar observations were made when detecting Rad51, a marker for homologous recombination (Fig. 8).

### Repair of DSBs is organised differently in *Nipbl*^*+/−*^ spermatocytes

**Fig. 9:**
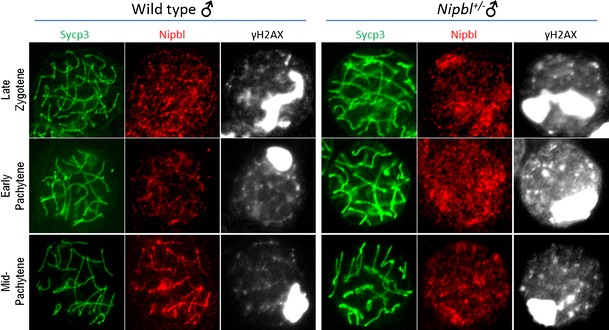
Pachytene *Nipbl* ^*+/−*^ spermatocytes display diffuse and disorganised staining of γH2AX. Immunofluorescent staining of Sycp3 (*green*), Nipbl (*red*) and γH2AX (*white*). Images were staged according to the distribution of Sycp3 and Nipbl along chromosomal axes. In late zygotene and early pachytene (*top row*), most of the ubiquitous γH2AX of early prophase signals disappear from the nucleus, except at the sex body, which appears in as an irregular elongated shape. In wild type, one can observe residual γH2AX signals organised at chromosome axes, while the γH2AX outside of the sex body in *Nipbl* ^*+/−*^ are more prominent and distributed all over the nucleus. In early and mid-pachytene, when Nipbl starts to re-localise to chromocentres (*middle and bottom rows*), residual γH2AX staining is still stronger in *Nipbl* ^+/−^ than in wild type

When comparing the distribution of γH2AX during prophase I in spermatocytes from *Nipbl* ^*+/−*^ and wild-type mice, we observed interesting differences. In wild type, the pan-nuclear γH2AX staining seen in leptotene was gradually reduced, except at the sex body. Outside of the sex body, remnants of γH2AX were observed on chromosomal axes up to mid-pachytene, as previously reported (Chicheportiche et al. 2007). Interestingly, this was the same stage when Nipbl was lost from chromosomal axes. However, in *Nipbl* ^+/−^ spermatocytes, at the stage when the sex body is strongly stained by γH2AX and seen as an elongated structure, γH2AX was not seen organised at chromosomal axes (Fig. 9). Instead, γH2AX was found as seemingly disorganised foci throughout the nucleus, presumably associated with chromosome loops. In contrast to wild type, *Nipbl* ^*+/−*^ spermatocytes maintained these ‘residual’ loop-associated foci even after the mid-pachytene stage when Nipbl had translocated to chromocentres. After this stage, very little γH2AX was seen outside of the sex body in wild-type cells, while *Nipbl* ^*+/−*^ diplotene cells frequently showed a few foci, generally associated with chromosomal axes (not shown).

### Spatial and temporal connection between Nipbl localisation to heterochromatin and H3K9me3

**Fig. 10:**
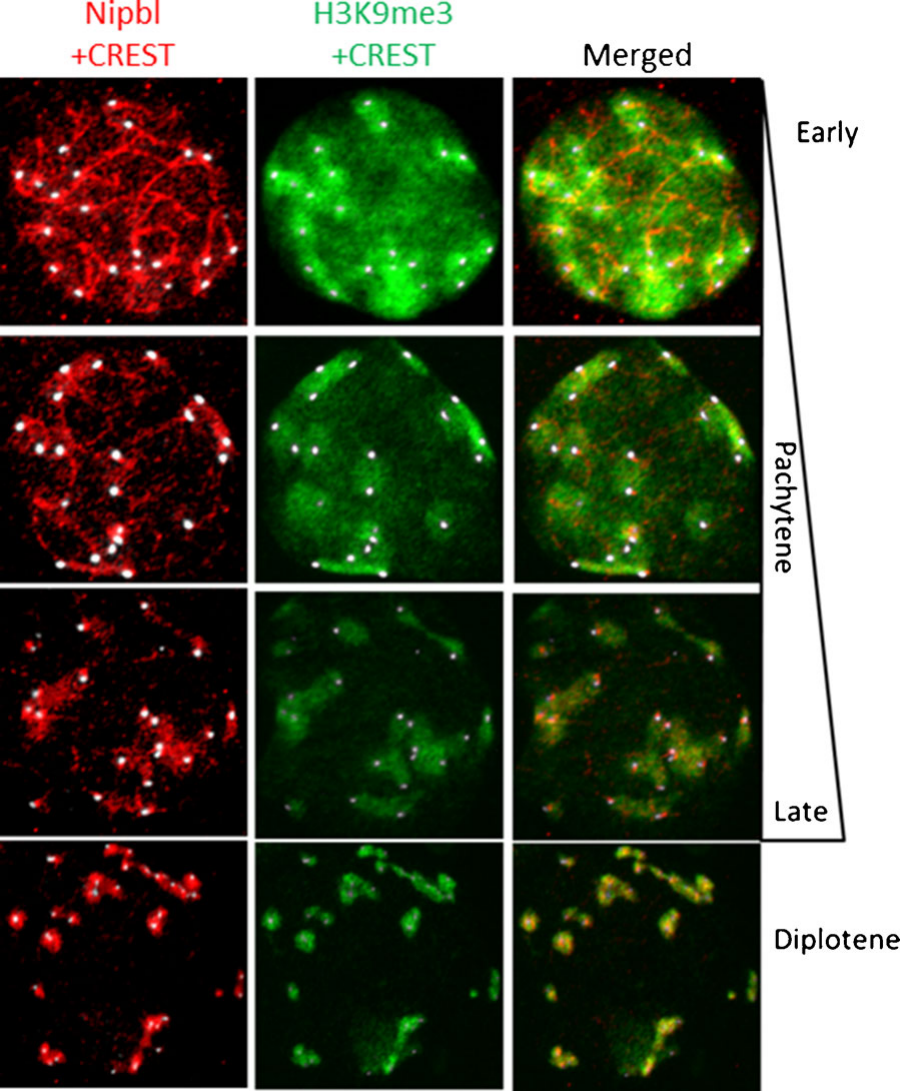
Spatial and temporal connection between Nipbl and H3K9me3 re-localisation to heterochromatin. Testicular nuclear spreads were stained with rabbit anti-H3K9me3 (*green*), guinea pig anti-Nipbl (*red*) and human anti-CREST (*white*). In early pachytene cells (*upper row*), H3K9me3 is seen on chromocentres and as a diffuse nuclear staining. During mid-pachytene (*middle rows*), H3K9me3 persists on chromocentres, while the diffuse nuclear staining fades concomitantly as Nipbl relocates to heterochromatin. In diplotene, both Nipbl and H3K9me3 can only be detected on chromocentres (*bottom row*)

To further characterise the translocation of Nipbl/Mau2 from chromosomal axes to chromocentres, observed in both wild-type and *Nipbl* ^*+/−*^ spermatocytes, we stained for histone 3 trimethylated at lysine 9 (H3K9me3), a recognised marker for heterochromatin. In leptotene, zygotene and early pachytene nuclei, we could detect H3K9me3 as a diffuse pan-nuclear staining with some accumulation at chromocentres (data not shown; Fig. 10). By mid-pachytene, both H3K9me3 and Nipbl/Mau2 appeared at chromocentres. Concurrently, while Nipbl/Mau2 relocated from axes to chromocentres, H3K9me3 staining outside of chromocentres was markedly reduced. Since we could not observe any apparent difference between H3K9me3 in wild-type and *Nipbl* ^*+/−*^ spermatocytes, recruitment of H3K9me3 to chromocentres appears to be insensitive to *Nipbl* gene dosage (results not shown).

## Discussion

In this study, we have determined the chromosome localisation of the cohesin loading complex Nipbl/Mau2 during meiotic prophase I in wild-type and *Nipbl* ^*+/−*^ mouse germ cells. We found that the complex binds to meiotic chromosome axis from zygotene to mid-pachytene. In developing oocytes, Nipbl/Mau2 remained bound to the chromosomal axes at least to dictyate arrest at E19.5, whereas in spermatocytes, the complex re-located to chromocentres during mid-pachytene, where it remained bound throughout the first meiotic prophase. In spermatocytes, we could observe significant overlap in staining between cohesin, condensin I and Nipbl/Mau2 during zygotene, consistent with a role for Nipbl/Mau2 as a loader of these complexes. After mid-pachytene, however, there was little overlap in staining indicating that when at chromocentres, Nipbl/Mau2 does not load detectable levels of neither cohesin nor condensin I. We could detect Smc5/6 at chromocentres as early as zygotene, indicating that this association was independent of Nipbl/Mau2. All three SMC complexes appeared rather insensitive to Nipbl gene dosage, as there were no detectable differences in staining between wild-type and *Nipbl* ^*+/−*^ spermatocytes. While Nipbl/Mau2 did not associate with sites of ongoing DSB repair, distribution of γH2AX appeared disorganised in *Nipbl* ^*+/−*^ pachytene spermatocytes.

Interestingly, we found a clear sexual dimorphism in meiotic prophase in that Nipbl/Mau2 bound to chromocentres during mid/late pachytene and diplotene in male germ cells, but remained stably associated with chromosomal axes in developing oocytes. It is tempting to speculate that during oogenesis, Nipbl/Mau2 is needed to maintain cohesin binding and cohesion during dictyate, a state that could last for months. In mid-pachytene spermatocytes, where the first meiotic division is only days away, the bulk of Nipbl/Mau2 complexes may no longer be required to load cohesin at axes and is removed or relocated to chromocentres. In support of our data, a nearly identical staining pattern for Nipbl in both male and female meiocytes, using two different antibodies towards Nipbl, was reported during the time of revision for this study (Kuleszewicz et al. 2013).

Taken together, our data are consistent with a role for Nipbl/Mau2 as a loader of cohesin in leptotene/zygotene. However, since Nipbl is relocalised to chromocentres in mid-pachytene, it appears that in spermatocytes, cytologically detectable levels of Nipbl are not required to maintain cohesin at chromosomal axes. The observation that meiotic cohesin subunits formed cytologically recognisable axes before its loading complex may suggest that cohesin is loaded independently of Nipbl/Mau2. Alternatively, and in our opinion more likely, this can be explained by a model where Nipbl/Mau2 loads cohesin at distinct loading positions, from which cohesin may slide along DNA in an ATP-dependent manner (Hu et al. 2011).

When comparing wild-type and *Nipbl* ^*+/−*^ spermatocytes, no difference in staining of any SMC complex subunit investigated was found. This indicates that the chromatin association for the majority of SMC complexes is normal even though *Nipbl* gene dosage is reduced. Indeed, recent evidence suggests that the cohesin loading defect in *Nipbl* ^*+/−*^ cells is limited to a few specific loci (Remeseiro et al. 2013). It is possible that the methods employed in this paper are not sensitive enough to detect this type of subtle loading defects, and it is also conceivable that any putative loading defect of condensin or Smc5/6 is restricted to only a few discrete loci as well. That said, the staining patterns observed might still be informative. For instance, while it is hard to reconcile our data with a role for Nipbl/Mau2 as a loading complex for Smc5/6, they are consistent with a potential role for Nipbl/Mau2 as a loader for condensin I, since we do not see condensin I at regions that are not previously bound by Nipbl/Mau2. As for cohesin, the highest degree of co-localisation was observed in zygotene, where both Nipbl/Mau2 and condensin I covered extensive stretches of chromosomal axes. In pachytene and later stages, condensin I was to a large degree removed from chromosomal axes, but retained at chromosome ends and at the sex body. Somewhat confusing, this localisation pattern is partially inconsistent with that reported for the kleisin subunit of condensin I, Cap-H (Viera et al. 2007). While both reports observe condensin I at chromosome ends during diplotene, we also observe condensin I localising to chromosomal axes as early as zygotene. A possible explanation for this could be that different antibodies to Cap-G were employed with slightly different epitopes. Alternatively, variations in accessibility of antibody epitopes when applying different methods for fixation, extraction and staining could also account for the different localisation patterns during zygotene and pachytene.

Hence, even though SMC complexes mediate extensive chromosomal rearrangements during meiosis, their localisation appears unaffected by the type of partial NIPBL insufficiency that induces DNA repair defects and gene expression dysregulation in CdLS. This is also consistent with data from other organisms, where expression of the loading complex must be reduced to near completion to cause cohesion and segregation defects (Heidinger-Pauli et al. 2010). We could also show that the radiation sensitivity observed in MEFs haploinsufficient for Nipbl was comparable to that observed for CdLS cells (Vrouwe et al. 2007; Enervald et al. 2013). Although this sensitization is believed to be caused by defects in both HR and classical non-homologous end joining, even at very low radiation doses, and that meiosis is dependent on an efficient HR machinery, we could not observe any DNA repair defect in *Nipbl* ^*+/−*^ spermatocytes per se. Instead, γH2AX clearance was organised differently in *Nipbl* ^*+/−*^ spermatocytes, taking place to a higher degree on chromosomal loops instead of along chromosomal axes. Interestingly, spermatocytes with a lower expression of the meiosis-specific subunit SMC1β display multiple irregular γH2AX foci throughout the nucleus in the later phases of prophase (Murdoch et al. 2013), similar to the observations made here. Thus, this aspect of DSB repair may be extremely sensitive to the dosage of cohesin or its loading complex. Our observations that Nipbl/Mau2 predominantly binds outside of repair foci seem to strengthen the recent ChIP-based observation that cohesin binds at the border of restriction-enzyme induced γH2AX foci in a human cancer cell line (Caron et al. 2012), perhaps limiting the spread of γH2AX along chromosomes.

This altered organisation of γH2AX was, however, clearly insufficient to induce DSB repair defects on the same scale as in mitotic cells, suggesting that meiotic cells can protect themselves against the DNA repair defect caused by *Nipbl* haploinsufficiency. While most tested *Nipbl* ^*+/−*^ males were fertile, 4 out of 14 males (28.5 %) bred for at least 2 months were unable to sire a single litter. In average, 14.2 % of the weaned pups were *Nipbl* ^*+/−*^ (*n* = 366). This is consistent with the post-natal lethality suggested by Kawauchi et al. (2009), who reported that about half of *Nipbl* ^*+/−*^ pups died between conception and weaning. However, there were substantial and consistent variations between male breeders, with *Nipbl* ^*+/−*^ pups varying from >40 % *Nipbl* ^*+/−*^ (*n* = 1), 30–40 % (*n* = 3), 20–30 % (*n* = 3), 10–20 % (*n* = 3) and <10 % (*n* = 3). Taken together, our data suggests that the *Nipbl* ^*+/−*^ males are largely fertile, and that the failure of some males to sire pups may rather be due to physical or behavioural alterations.

Interestingly, the strong co-localisation between H3K9me3 and Nipbl that we found in the later stages of prophase I appeared to be independent of cohesin or any other SMC complex. While unexpected, this is not without support in the literature. Nipbl interacts with heterochromatin protein 1 gamma (HP1γ) through its PxVxL motif (Lechner et al. 2005) and has also been shown to interact with several histone deacetylases (Jahnke et al. 2008). Since the overall intensity of H3K9me3 staining per nucleus was similar during all stages of the meiotic prophase (results not shown), this suggests that the accumulation of H3K9me3 at pericentromeric heterochromatin is not controlled by the activity of histone deacetylases, which would presumably be important to promote heterochromatinisation of histones. The function of Nipbl/Mau2 at these regions is currently not known. In principle, binding to heterochromatin might inhibit, activate or be unrelated to the cohesin loading activity of Nipbl/Mau2. Since MEFs lacking the Suv39h1 methyltransferases do not show any defect in cohesin loading, at pericentric heterochromatin or elsewhere (Koch et al. 2008), we find it unlikely that this interaction stimulates cohesin loading by Nipbl/Mau2. Instead, we are tempted to speculate that binding of Nipbl/Mau2 to heterochromatin may regulate the cohesin loading negatively. First, as far as we are aware, binding of cohesin to chromocentres has not been observed in mammalian meiosis. Second, Nozawa et al. (2010) searched for heterochromatin-interacting proteins using a proteomics-based approach and were able to identify both NIPBL and MAU2 among the top hits. However, except for the shugoshin protein, no other cohesin component was found. This suggests that Nipbl/Mau2 does not load cohesin when interacting with pericentric heterochromatin. This may either inhibit loading of cohesin to DNA or be important for other functions of the complex, an interesting topic for future investigations.

## Electronic supplementary material

Below is the link to the electronic supplementary material.

Supplementary Fig. 1 Validation of guinea pig anti-Nipbl and anti-Mau2 and rabbit anti-Cap-G antibodies. Western blot of wild-type and *Nipbl* ^*+/−*^ E13.5 mouse embryonic fibroblasts detecting Nipbl (A), Mau2 (B) and Cap-G (C). The arrows indicate Nipbl, Mau2 or Cap-G bands of expected sizes. Staining of membranes with Poncau, before antibody detection, is used as control for equal protein levels between lanes. (JPEG 26 kb)

High resolution image (TIFF 202 kb)

Supplementary Fig. 2 Nipbl binds to centromeres and pericentric heterochromatin in post-prophase I stages. Testicular nuclear spreads were stained with rabbit anti-Sycp3 (red), guinea pig anti-Nipbl (green) and stained with DAPI (blue). **A** Metaphase I. Nipbl is detected at the centromeres co-localising with Sycp3, and as two separated pairs of dots, suggestive of centrioles (arrowheads). **B** Anaphase I. The Nipbl signals at centromeres become weaker at this stage but are still evident. A bright Nipbl staining is also evident at presumptive centrioles (arrowheads). **C** Interkinesis. The chromocentres are labelled by the Nipbl antibody. Inside chromocentres some brighter signals of Nipbl co-localise with Sycp3 bars typical of this stage. Putative centrioles are also observed as bright Nipbl signals (arrowhead). **D** Early round spermatid. One centrally located chromocentre presents a faint Nipbl labeling. **E** and **F** Elongated spermatids. A bright pair of Nipbl dots at the base of the spermatids are observed. (JPEG 69 kb)

High resolution image (TIFF 1194 kb)

Supplementary Fig. 3 Yeast Scc4 meiotic null cells are completely incapable of progressing through meiosis. **A** Western blot showing down-regulation of HA-tagged Scc4 after release into nitrogen depleted sporulation medium. Cdc11, which is stably expressed throughout meiosis, is used as loading control. **B** Seven hours after release into sporulation medium, wild-type cells undergo meiotic divisions, forming four daughter nuclei. A small portion of Scc4 meiotic null cells undergo the first meiotic division, forming two nuclei, but do not progress further. (JPEG 47 kb)

High resolution image (TIFF 193 kb)

Supplementary Fig. 4 Mau2 staining in *Nipbl* ^*+/−*^ spermatocytes. Top row: In zygotene nuclei of wild-type and *Nipbl* ^*+/−*^ spermatocytes, both rabbit anti-Mau2 (green) and guinea pig anti-Nipbl (red) bind to chromosomal axes. Bottom row: In both genotypes, both Mau2 and Nipbl accumulate at chromocentres. (JPEG 50 kb)

High resolution image (TIFF 399 kb)

Supplementary Fig. 5 Localization of meiosis-specific Stag3 is similar to wild-type in *Nipbl* ^*+/−*^ spermatocytes. Rabbit anti-Sycp3 (green), guinea pig anti-Stag3 (red) and human anti-ACA (white) were stained in wild-type and *Nipbl* ^*+/−*^ spermatocytes. Stag3 decorates the chromosomal axes to a similar extent in both wild-type and *Nipbl* ^*+/−*^ spermatocytes. (JPEG 107 kb)

High resolution image (TIFF 834 kb)

Supplementary Fig. 6 Nipbl does not co-localise with γH2AX in irradiated mouse embryonic fibroblasts. E13.5 embryonic fibroblasts were fixed and stained with anti-Nipbl (red) and anti-γH2AX (green) before and 60 minutes after being exposed to 1 Gy ionizing radiation. (JPEG 40 kb)

High resolution image (TIFF 299 kb)
