# Supplementary figures and images for: Localisation of the SMC loading complex Nipbl/Mau2 during mammalian meiotic prophase I
Source: Chromosoma. 2013 Nov 28;123(3):239–52. doi: 10.1007/s00412-013-0444-7 (PMC4031387; doi:10.1007/s00412-013-0444-7)

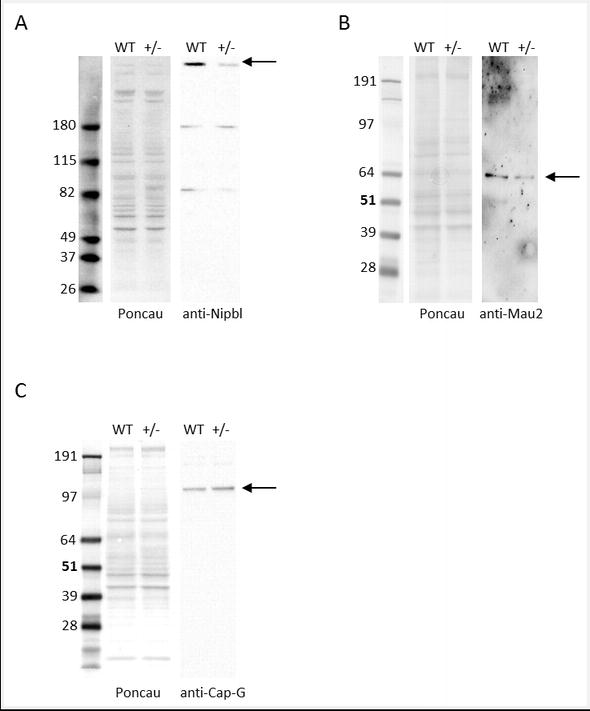

Supplement: Supplementary file 1 — Validation of guinea pig anti-Nipbl and anti-Mau2 and rabbit anti-Cap-G antibodies. Western blot of wild-type and Nipbl +/− E13.5 mouse embryonic fibroblasts detecting Nipbl (A), Mau2 (B) and Cap-G (C). The arrows indicate Nipbl, Mau2 or Cap-G bands of expected sizes. Staining of membranes with Poncau, before antibody detection, is used as control for equal protein levels between lanes. (JPEG 26 kb) [file 412_2013_444_Fig11_ESM.jpg]

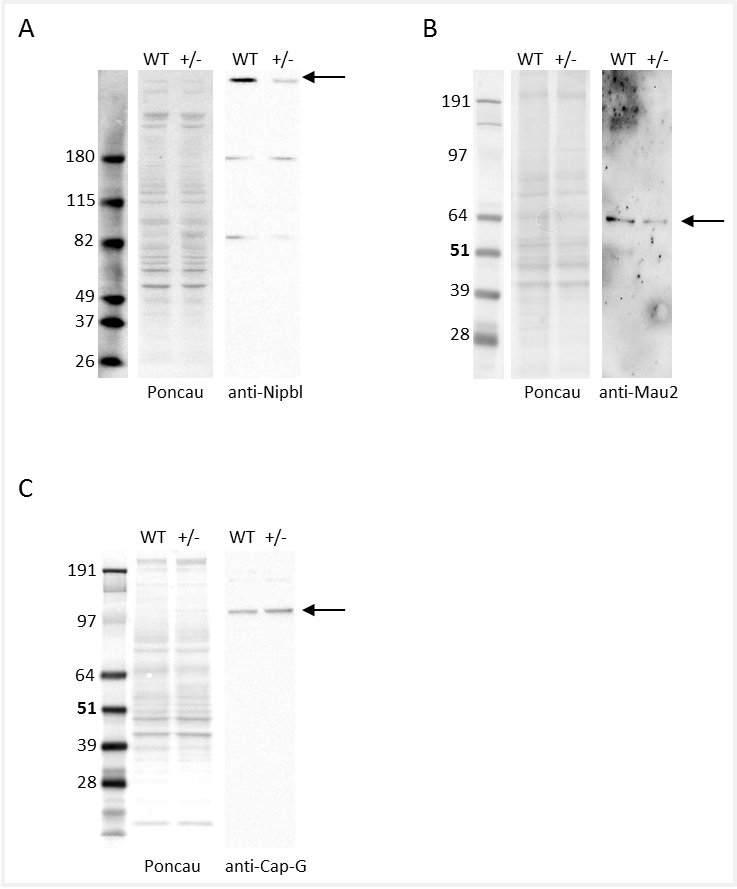

Supplement: Supplementary file 2 — High resolution image (TIFF 202 kb) [file 412_2013_444_MOESM1_ESM.tif]

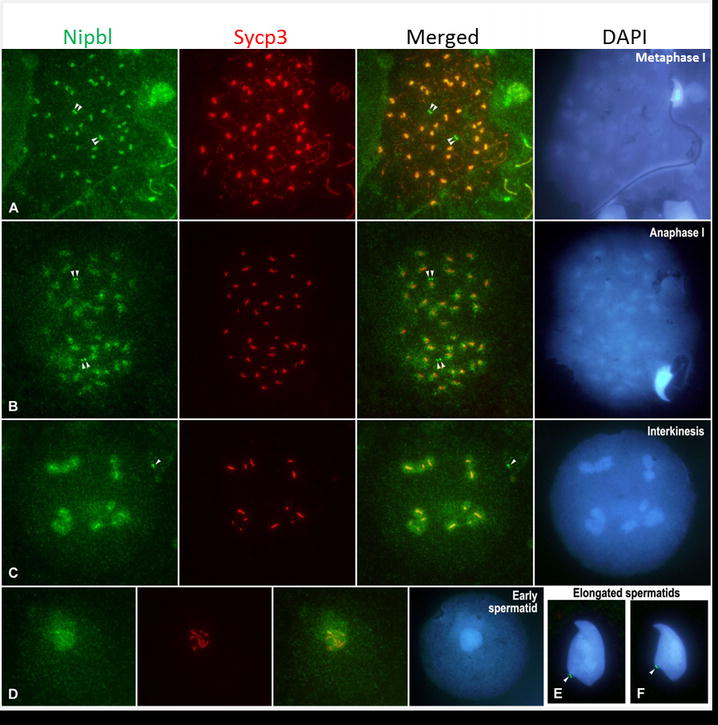

Supplement: Supplementary file 3 — Nipbl binds to centromeres and pericentric heterochromatin in post-prophase I stages. Testicular nuclear spreads were stained with rabbit anti-Sycp3 (red), guinea pig anti-Nipbl (green) and stained with DAPI (blue). A Metaphase I. Nipbl is detected at the centromeres co-localising with Sycp3, and as two separated pairs of dots, suggestive of centrioles (arrowheads). B Anaphase I. The Nipbl signals at centromeres become weaker at this stage but are still evident. A bright Nipbl staining is also evident at presumptive centrioles (arrowheads). C Interkinesis. The chromocentres are labelled by the Nipbl antibody. Inside chromocentres some brighter signals of Nipbl co-localise with Sycp3 bars typical of this stage. Putative centrioles are also observed as bright Nipbl signals (arrowhead). D Early round spermatid. One centrally located chromocentre presents a faint Nipbl labeling. E and F Elongated spermatids. A bright pair of Nipbl dots at the base of the spermatids are observed. (JPEG 69 kb) [file 412_2013_444_Fig12_ESM.jpg]

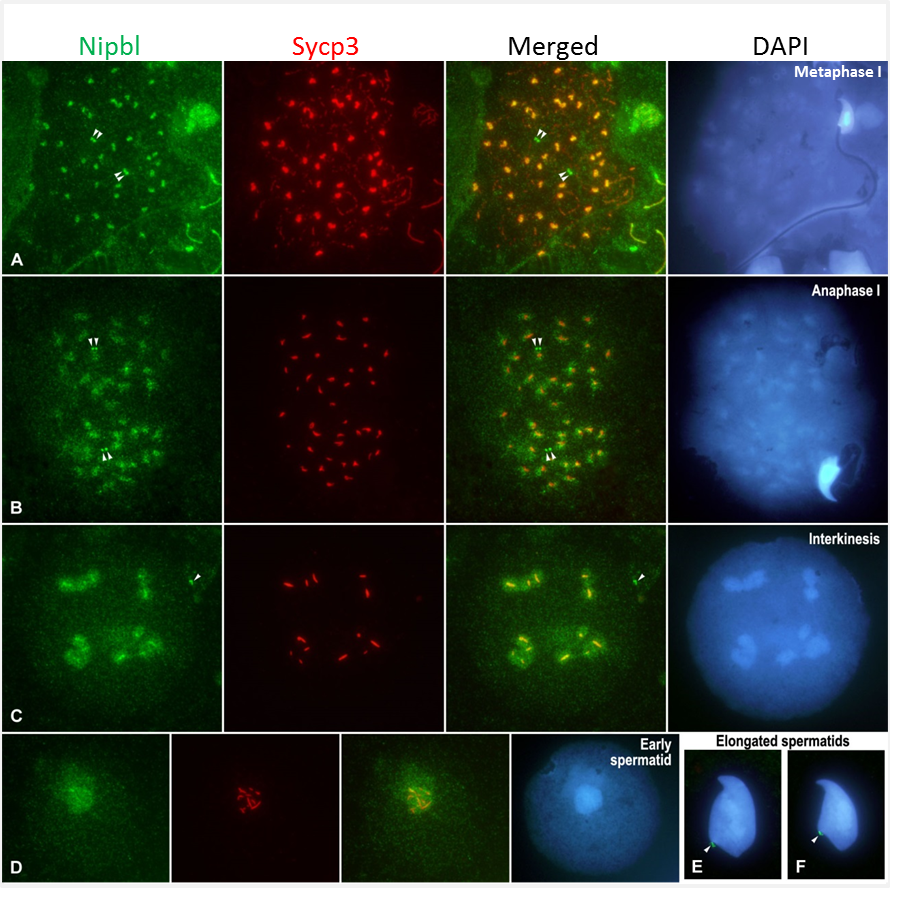

Supplement: Supplementary file 4 — High resolution image (TIFF 1194 kb) [file 412_2013_444_MOESM2_ESM.tif]

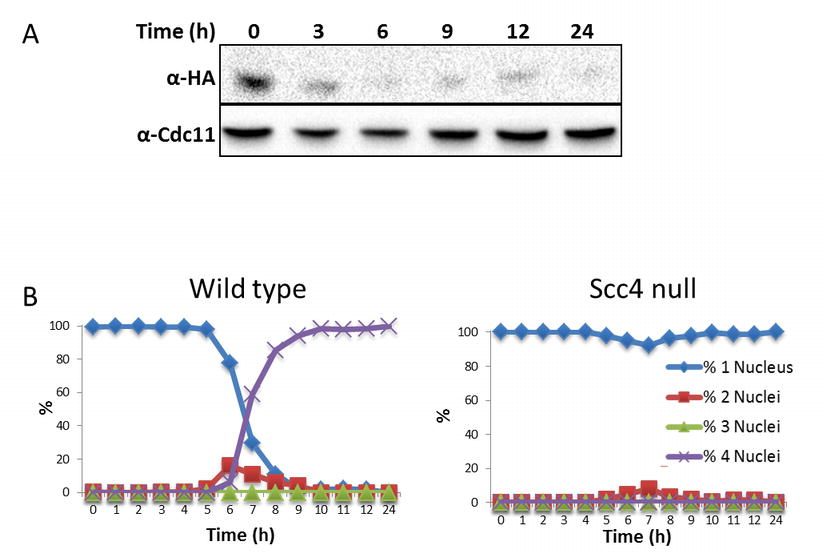

Supplement: Supplementary file 5 — Yeast Scc4 meiotic null cells are completely incapable of progressing through meiosis. A Western blot showing down-regulation of HA-tagged Scc4 after release into nitrogen depleted sporulation medium. Cdc11, which is stably expressed throughout meiosis, is used as loading control. B Seven hours after release into sporulation medium, wild-type cells undergo meiotic divisions, forming four daughter nuclei. A small portion of Scc4 meiotic null cells undergo the first meiotic division, forming two nuclei, but do not progress further. (JPEG 47 kb) [file 412_2013_444_Fig13_ESM.jpg]

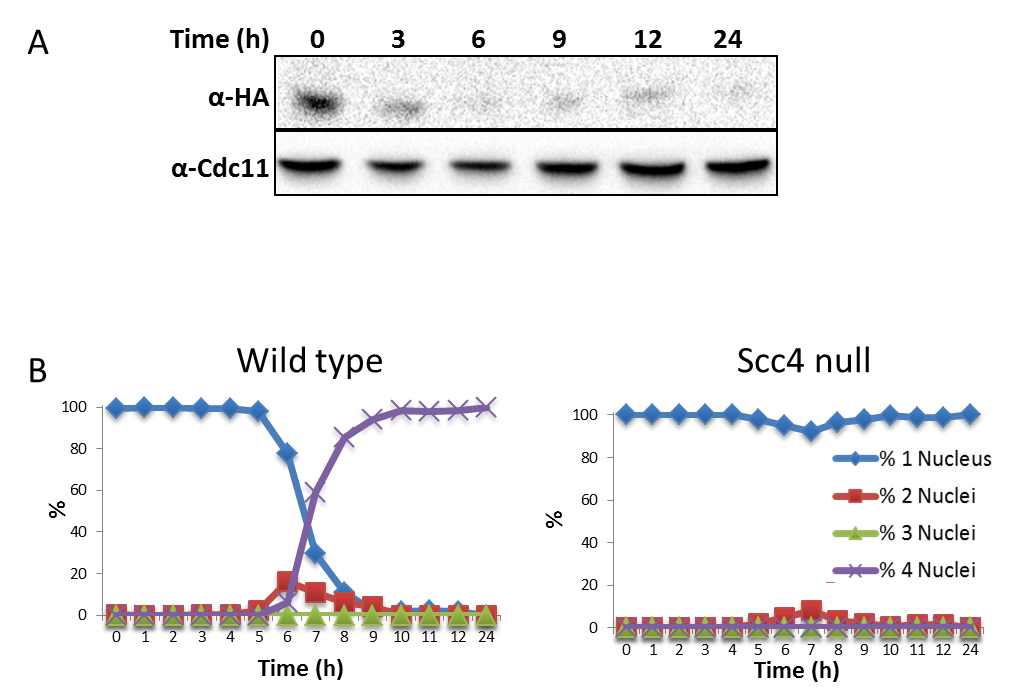

Supplement: Supplementary file 6 — High resolution image (TIFF 193 kb) [file 412_2013_444_MOESM3_ESM.tif]

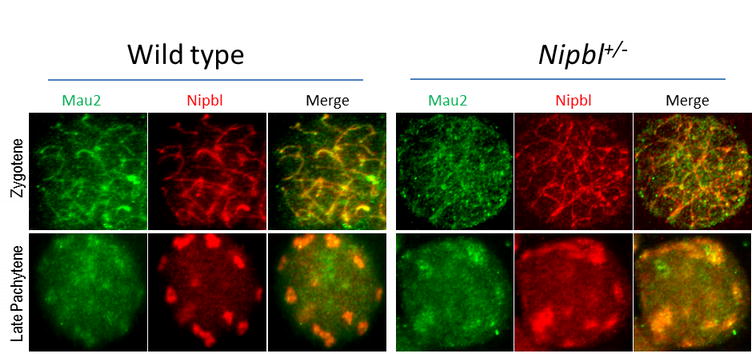

Supplement: Supplementary file 7 — Mau2 staining in Nipbl +/− spermatocytes. Top row: In zygotene nuclei of wild-type and Nipbl +/− spermatocytes, both rabbit anti-Mau2 (green) and guinea pig anti-Nipbl (red) bind to chromosomal axes. Bottom row: In both genotypes, both Mau2 and Nipbl accumulate at chromocentres. (JPEG 50 kb) [file 412_2013_444_Fig14_ESM.jpg]

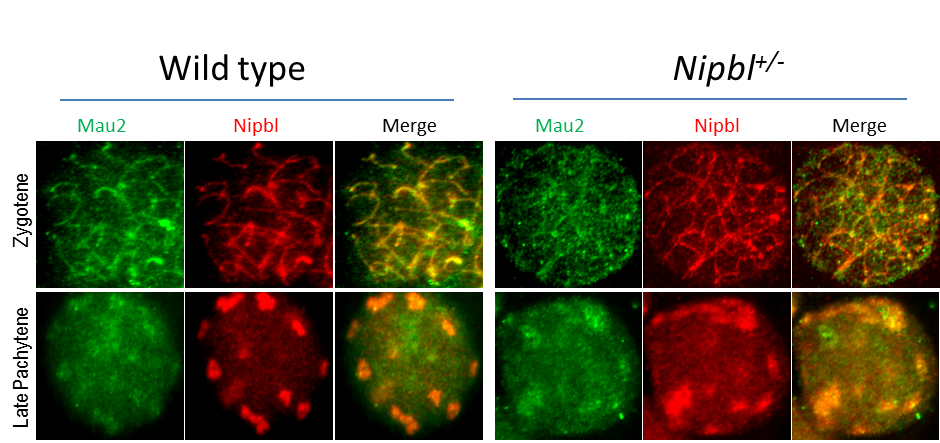

Supplement: Supplementary file 8 — High resolution image (TIFF 399 kb) [file 412_2013_444_MOESM4_ESM.tif]

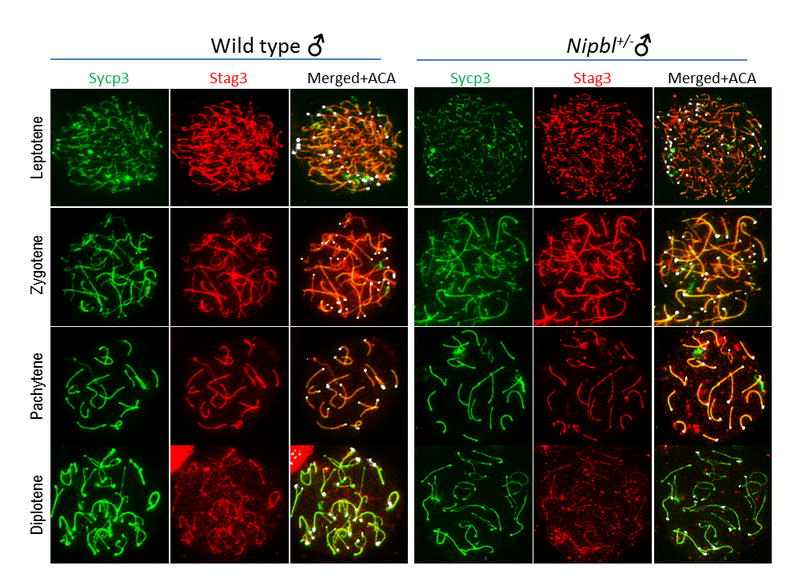

Supplement: Supplementary file 9 — Localization of meiosis-specific Stag3 is similar to wild-type in Nipbl +/− spermatocytes. Rabbit anti-Sycp3 (green), guinea pig anti-Stag3 (red) and human anti-ACA (white) were stained in wild-type and Nipbl +/− spermatocytes. Stag3 decorates the chromosomal axes to a similar extent in both wild-type and Nipbl +/− spermatocytes. (JPEG 107 kb) [file 412_2013_444_Fig15_ESM.jpg]

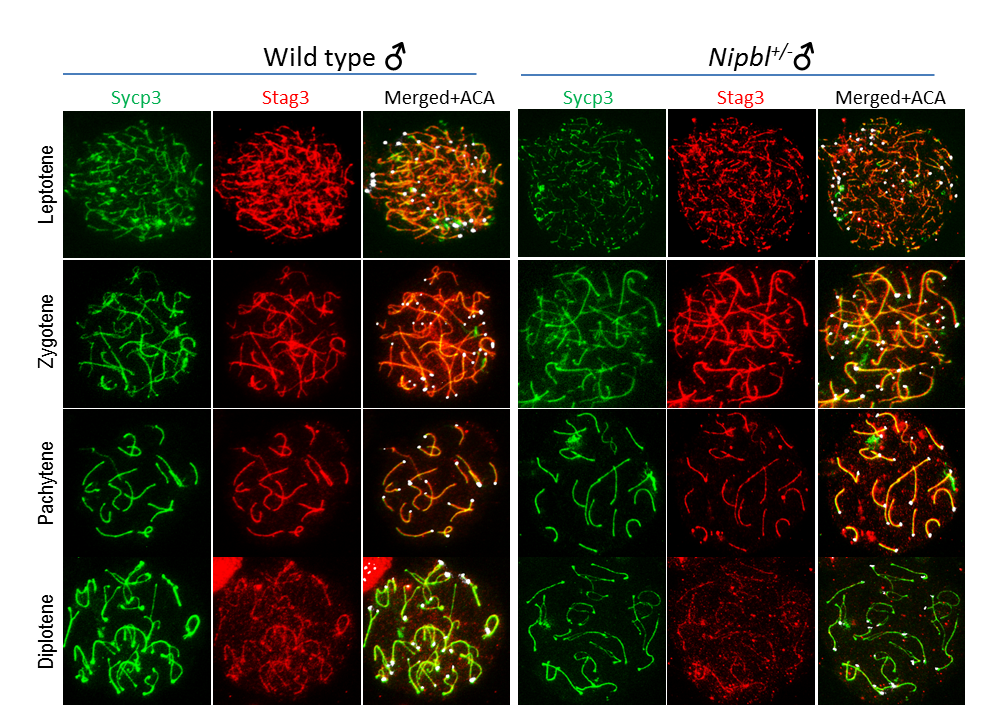

Supplement: Supplementary file 10 — High resolution image (TIFF 834 kb) [file 412_2013_444_MOESM5_ESM.tif]

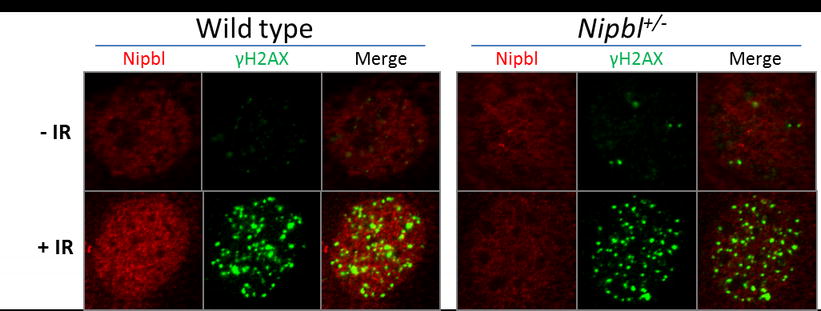

Supplement: Supplementary file 11 — Nipbl does not co-localise with γH2AX in irradiated mouse embryonic fibroblasts. E13.5 embryonic fibroblasts were fixed and stained with anti-Nipbl (red) and anti-γH2AX (green) before and 60 minutes after being exposed to 1 Gy ionizing radiation. (JPEG 40 kb) [file 412_2013_444_Fig16_ESM.jpg]

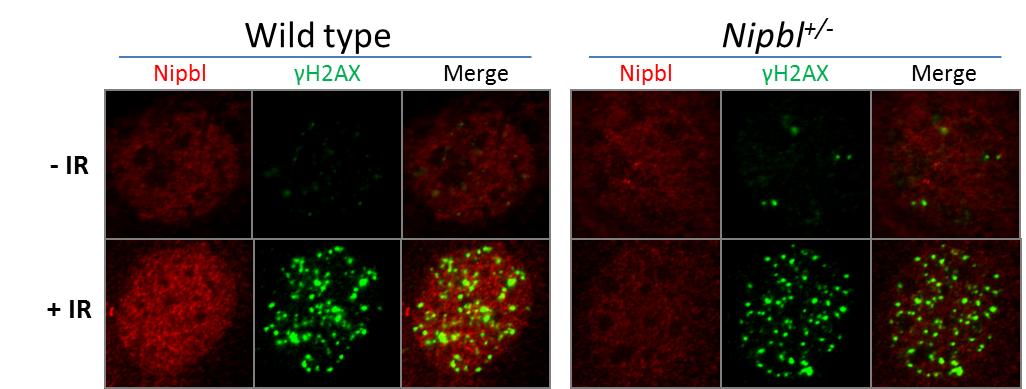

Supplement: Supplementary file 12 — High resolution image (TIFF 299 kb) [file 412_2013_444_MOESM6_ESM.tif]
